# Tackling challenges of TB/MDRTB in China: concerted actions are imperative

**DOI:** 10.1186/s40249-015-0050-4

**Published:** 2015-04-16

**Authors:** Shenglan Tang

**Affiliations:** Duke Global Health Institute, Duke University, Durham, USA; Duke Kunshan University, Global Health Research Center, Kunshan, China

**Keywords:** Tuberculosis, China

## Abstract

China is the second largest TB epidemic with the most number of people infected with multi-drug resistant (MDR). Over the past decade, a large number of TB control projects have been funded by the government of China and international organizations, and epidemic in China has been effectively controlled. However TB control in China still faced many challenges. Strategies to address these challenges may include integrating the national TB control program into health insurance schemes, strengthening TB case management through involving the Chinese hospital in national TB control program, and reforming payment methods for TB care as part of health system reform in China.

**Electronic supplementary material:**

The online version of this article (doi:10.1186/s40249-015-0050-4) contains supplementary material, which is available to authorized users.

## Multilingual abstracts

Please see Additional file 1 for translations of the abstract into the six official working languages of the United Nations.

## Background

Worldwide tuberculosis (TB) remains a leading cause of death. It was estimated 9.0 million people developed TB and 1.5 million died from the disease, 360,000 of whom were HIV-positive in 2013 [1]. Globally, the proportion of new cases with multidrug-resistant TB (MDR-TB) was 3.5% in 2013 and has not changed compared with recent years [2]. China is the second largest TB epidemic with the most number of people infected with multi-drug resistant (MDR) [1-3]. About 5.7% of newly diagnosed TB cases, and 25.6% of previously treated TB cases in China are multi-drug resistant [3]. Dr Li and his colleagues reported that those being single, without health insurance coverage, with higher life pressure, and from low income group are high risk factors associated with MDRTB [4]. A systematic review published recently claimed that delay in being treated increases the risk of spreading TB, and contributes to the development of MDR-TB. Patients who delayed going to see a doctor were more likely to live in rural areas, to be less educated or female [5]. Poverty, lack of health insurance, rising costs of excess payments or treatments not covered by the insurance, as well as a poor understanding of TB were cited by patients in contributing to their delay in seeing a doctor. There is also a stigma associated with TB as reported widely.

## Main text

Over the past decade, a large number of TB control projects have been funded by the government of China, the World Bank, Department for International Development/UK, and the Global Fund to Fight HIV/AIDS, TB and Malaria, with a significant amount of financial resources invested. As a consequence of these projects, TB epidemic in China has been effectively controlled [6]. The National TB Epidemiological Survey conducted in 2010 shows that significant progress has been made to control TB, including a remarkable reduction in smear- and culture-positive TB prevalence rates and fewer TB patients are failing to complete the treatment because of financial difficulty [6]. However, the prevalence rate of MDR-TB has changed very little over the past decade. The active TB prevalence rate in rural areas, particularly in the western part of rural China, has actually increased. Among 1,301 active TB patients detected in the survey, the proportion of the patients diagnosed prior to the survey rose from 69% in 2000 to 90% in 2010 [6]. Such a result may be due in part to more TB patients without any reported symptoms. Or this may imply that the TB case detection in China has not improved, despite large investments in TB control by the Chinese government and the international community. Of 740 newly-diagnosed TB patients with at least one reported symptom in the 2010, 53% of them had never sought any professional care, an increase from 42% in 2000 [6]. At the same time, health insurance coverage rose from 35% in 2000, to 95% in 2010 [6]. This could indicate that the health insurance schemes, especially operated in rural China, have not provided sufficient financial protections to those in needs, as often the health insurance policies do not cover outpatient services. Among the newly- diagnosed TB patients, the average health care expenditure per patient was 1,708 Chinese yuan, of which 84% of the expenses were paid out of pocket, although a vast majority of them are covered by the “new rural cooperative medical scheme (NRCMS)” [7]. Among those 346 patients with TB-related symptoms, only 124 patients (36%) were diagnosed as TB cases before the 2010 survey [6]. The TB case detection rate among the same patient group declined from 61% in 2000 to 36% in 2010, a seriously alarming finding [6]. This illustrates that the quality of TB care has not improved, and may have worsened in some health facilities.

What should be done? These problems clearly indicate that concerted actions are badly needed in order to tackle the challenge of TB/MDR-TB facing China. The Chinese health policy makers and TB control program managers should develop new strategies that can be more cost-effective and implemented to achieve the goal of eliminating TB by 2050, as advocated by WHO in recent years [8]. I believe following action would help the national TB control program to make a greater progress in fighting against TB/MDR-TB.

It is critically important to integrate the national TB control program into the health system development, especially health insurance schemes. However, it is essential, but not sufficient, as the health insurance policies often require a co-payment [9], which may be a financial barrier for TB patients to get their illnesses diagnosed timely, and for diagnosed TB patients to complete their due treatments. TB is an infectious disease of poverty, often affecting the poor and vulnerable, as reported by many studies. A modest deductible or co-insurance payment could be a financial burden placed on their households. Hence, the national TB control program should also work with the Ministry of Civil Affairs that is responsible for medical financing assistance for the poor to ensure that TB patients, particularly MDR-TB patients, can be diagnosed timely and then treated successfully, regardless of their ability to pay for health care. It is good to see that with the support from the Gates Foundation, China CDC has tried to develop such concerted actions to reduce financial burden of TB/MDR-TB care placed on the patients.

The national TB control program, as part of China CDC system, should also closely work with the Chinese hospital sector to strengthen TB case management, particularly in the rural areas. There should be clear clinical guidelines for treatment and effective case management put in place. It is also crucial to develop monitoring and evaluation systems to assess the performance of these so-called TB designated hospitals.

Paying for TB care should also be seen as part of the health system reform in China [10]. This can be accomplished by using alternative provider payment methods to fee-for-services, in order to remove perverse financial incentives and reduce moral hazards of the Chinese hospitals, the majority of which have been maximizing their profits from service provision and drug sale over the past 2–3 decades. Without effective cost containment measures, more financial resources for TB control and care might be wasted, let alone being able to produce the health gains that the government is expecting.

## Conclusion

Strategies to address current challenges in China need to integrate the national TB control program into health insurance schemes, strengthening TB case management through involving the Chinese hospital in national TB control program, and reforming payment methods for TB care as part of health system reform in China.

## Additional file

Additional file 1:
**Multilingual abstracts in the six official working languages of the United Nations.**
