# Supplementary material for: Tackling challenges of TB/MDRTB in China: concerted actions are imperative
Source: Infect Dis Poverty. 2015 Apr 16;4:19. doi: 10.1186/s40249-015-0050-4 (PMC4407541; doi:10.1186/s40249-015-0050-4)
Supplement: Additional file 1: — Multilingual abstracts in the six official working languages of the United Nations. [file 40249_2015_50_MOESM1_ESM.doc]

**中国迎接结核病/耐多药结核病的挑战:急需采取多种行动**

汤胜蓝

**摘要:**

中国是世界上第二大结核病高负担国,也是世界上耐多药结核高负担国。在过去十年中,中国政府和世界相关基金资助了大量的结核病防控项目,中国结核病控制取得了很大进展。但是中国结核病防控仍面临众多挑战。中国应对这些挑战需要采取多种行动,包括将国家结核病防控纳入医疗保险,在国家结核病防控规划中加强医院在结核病治疗管理中的作用,结核病诊疗的支付方式的改革纳入当前医疗卫生体制改革。

Translated from English version into Chinese by Yang Pin
